# Racial, Ethnic, and Socioeconomic Survival Disparities in Early-Onset Metastatic Colorectal Cancer

**DOI:** 10.1001/jamanetworkopen.2025.53146

**Published:** 2026-01-08

**Authors:** Jennifer S. Wang, Benny Johnson, Caitlin C. Murphy

**Affiliations:** 1 Department of Epidemiology, UTHealth Houston School of Public Health, Houston, Texas; 2 Department of Gastrointestinal Medical Oncology, University of Texas MD Anderson Cancer Center, Houston; 3 Now with Agenus Inc, Lexington, Massachusetts; 4 Department of Health Promotion & Behavioral Sciences, UTHealth Houston School of Public Health, Houston, Texas; 5 Now with Department of Pediatrics, University of Chicago, Chicago, Illinois

## Abstract

**Importance:**

Rates of metastatic colorectal cancer (mCRC) are rising among young adults. Disparities by race and ethnicity and neighborhood-level socioeconomic status (SES) among this population are understudied.

**Objective:**

To examine the association of race and ethnicity and neighborhood-level SES with mortality among a community-based sample of young adults with mCRC.

**Design, Setting, and Participants:**

This cohort study used a large electronic health record–derived database of young adults with cancer treated at 280 community-based US clinics between 2013 and 2021. Eligible patients were young adults aged 18 to 49 years diagnosed with de novo or recurrent mCRC. Patients were followed up until December 31, 2022. Data were analyzed from February 2024 to November 2025.

**Exposures:**

Race and ethnicity and neighborhood-level SES. Neighborhood-level SES was derived using census block group (2010 Census boundaries) and most recent address in the electronic health record. Five-year estimates from the American Community Survey (2015-2019) were used to compute the Yost Index, a composite measure of 7 variables that capture different aspects of neighborhood-level SES.

**Main Outcome and Measure:**

All-cause mortality and 1-, 2-, and 3-year survival from diagnosis. Survival was defined from date of de novo or recurrent mCRC diagnosis to date of death or December 31, 2022.

**Results:**

A total of 3115 young adults diagnosed with mCRC (mean [SD] age at diagnosis, 42.4 [5.9] years; 122 Asian [3.9%], 424 Black [13.6%], 395 Hispanic [12.7%], 1874 White [60.2%]; 1651 male [53.0%]). Survival differed significantly by race and ethnicity and neighborhood-level SES. At 3 years after diagnosis, Black patients had worse survival (41%; 95% CI, 36%-46%), while Asian (58%; 95% CI, 48%-66%) and Hispanic (53%; 95% CI, 48%-58%) patients had better survival compared with White patients (47%; 95% CI, 45%-49%). For neighborhood-level SES, 3-year survival was 41% (95% CI, 36%-45%) for patients in the lowest compared with 59% (95% CI, 54%-63%) in the highest quintile. In adjusted analyses, neighborhood-level SES was associated with mortality (Q1 vs Q5: hazard ratio [HR], 1.51; 95% CI, 1.24-1.82), while the HR for Black race and mortality was greater than 1 but not statistically significant (HR, 1.08; 95% CI, 0.90-1.31).

**Conclusions:**

In this cohort study of young adults with mCRC, 3-year survival differed by race and ethnicity and neighborhood-level SES, but only the association between neighborhood-level SES and survival remained statistically significant after adjusting for covariates.

## Key Points

**Question:**

Are race and ethnicity and neighborhood-level socioeconomic status (SES) associated with mortality among young adults with metastatic colorectal cancer (mCRC)?

**Findings:**

In this cohort study of 3115 young adults with mCRC, 3-year survival was lower among Black patients (40.7%) and patients in the lowest SES quintile (40.6%). Patients living in the lowest SES quintile had 51% higher risk of mortality, and Black patients had 8% higher risk of mortality, although the latter association was not statistically significant after adjusting for sociodemographic, tumor, and treatment characteristics.

**Meaning:**

These findings suggest that structural conditions, such as neighborhood-level SES, impact survival among young adults with mCRC.

## Teaser

This cohort study of US young adults with metastatic colorectal cancer (mCRC) examines the association of race, ethnicity, and socioeconomic status with mortality.

## Introduction

Over the past 3 decades, there has been an alarming increase in incidence rates of colorectal cancer among young adults (age 18 to 49 years) that contrasts declining rates among older adults. Incidence rates of metastatic colorectal cancer among young adults (hereafter *early-onset mCRC*), in particular, has increased by 22% between 2010 and 2019, from 3.3 to 4.0 per 100 000.^1^ As a consequence, colorectal cancer is now the leading cause of cancer death in young men and second leading cause of cancer death in young women in the US.^2^

Colorectal cancer disproportionately affects historically minoritized racial and ethnic groups.^3,4^ For example, Black individuals—across all age groups—have higher incidence rates of colorectal cancer and worse relative survival compared with White individuals.^3,5^ Although Hispanic individuals have lower incidence rates of colorectal cancer, they also have worse survival compared with White individuals.^6^ Neighborhood-level socioeconomic status (SES), variously defined as the economic, social, and physical conditions of a neighborhood, has also been associated with higher incidence rates of and worse survival from colorectal cancer.^4,7,8,9^ For example, compared with the lowest poverty neighborhoods (measured at the census tract–level), incidence rates of colorectal cancer were greater in the highest poverty neighborhoods across 15 states.^9^ Socioeconomic disadvantage, defined by zip code–level area deprivation index, has similarly been associated with a 24% increased risk of mortality among older patients with colorectal cancer, even after adjusting for individual-level SES (hazard ratio [HR], 1.24; 95% CI, 1.17-1.32).^8^

Most studies of racial and ethnic or socioeconomic disparities in colorectal cancer have relied upon data from population-based cancer registries, such as the National Cancer Institute’s Surveillance, Epidemiology, and End Results (SEER) Program.^3,5^ Although these data are an important source of cancer information in the US, they overrepresent urban and foreign-born populations and lack detailed information on cancer treatment and tumor-related characteristics (eg, microsatellite instability, *BRAF* or *KRAS* variants). Importantly, the latter may differ across population groups and contribute to survival.^4,10,11^ Other studies have used data from a single center or an academic setting, limiting generalizability as most patients with cancer receive care in community-based settings.^12,13^

To address these gaps, we examined differences in survival related to racial, ethnic, and socioeconomic factors among a diverse, community-based sample of young adults diagnosed with mCRC. We overcome limitations of previous studies by using electronic health record–derived data with detailed information on cancer treatment and tumor-related characteristics from nearly 300 cancer clinics across the US. We uniquely focus on mCRC given well-documented delays in diagnosis among young adults and provide important insight into disparities by race and ethnicity and neighborhood-level SES among this growing but understudied population.^14^

## Methods

We used the nationwide Flatiron Health electronic health record–derived database, which includes deidentified data from over 280 community-based cancer clinics (800 sites of care) and represents more than 3.5 million patients diagnosed with cancer in the US. This longitudinal database includes patient-level structured and unstructured data curated via technology-enabled abstraction and machine learning methods. The study was approved by the institutional review board (IRB) at the University of Texas Health Science Center at Houston. The IRB waived the requirement for informed consent because all data from Flatiron Health are deidentified. We followed the Strengthening the Reporting of Observational Studies in Epidemiology (STROBE) reporting guidelines for cohort studies.

Our study population included young adults diagnosed with de novo or recurrent mCRC at age 18 to 49 years between January 1, 2013, and December 31, 2021. We combined concepts of race and ethnicity and included young adults who were non-Hispanic Asian, non-Hispanic Black, Hispanic, non-Hispanic White, and non-Hispanic other. Race and ethnicity were coded by Flatiron Health, with other including American Indian or Alaska Native, Hawaiian or Pacific Islander, and multiracial individuals. We excluded those missing race and ethnicity (405 [8.7%]), who did not initiate treatment (668 [14.5%]), or who initiated treatment more than 365 days after date of mCRC diagnosis (418 [9.1%]).

We described sociodemographic, tumor, and treatment characteristics of the study population by race and ethnicity and neighborhood-level SES and used χ^2^ or Fisher exact tests to assess differences. Neighborhood-level SES was derived using census block group (2010 Census boundaries) and based on the most recent address in the electronic health record. Flatiron Health used 5-year estimates from the American Community Survey (2015-2019) to compute the Yost Index, a composite measure of 7 variables that capture different aspects of neighborhood-level SES, such as median household income, median housing value, and poverty rate. Sociodemographic characteristics included age at mCRC diagnosis (18 to 29, 30 to 39, and 40 to 49 years), year of diagnosis, sex, insurance (private or commercial, Medicaid, Medicare, other government, uninsured, and other or unknown), and practice setting (academic, community). Tumor characteristics included diagnosis type (de novo or recurrent), location (colon or rectum), *RAS* variant (*NRAS* and/or *KRAS*), *BRAF* variant, microsatellite instability (mismatch repair deficiency and/or high microsatellite instability), and Lynch syndrome (pathogenic germline variant in *MLH1, MSH2, MSH6,* or *PMS2*). Treatment characteristics included clinical trial enrollment (any, none), time from diagnosis to treatment, and treatment regimen in the first- and second-line setting. Clinical trial enrollment was defined as receipt of a clinical study drug in any line of treatment.^15^ Each line of treatment was derived using Flatiron Health oncology-defined rules as a drug episode plus other eligible drugs given within 28 days of the start of treatment.^16^ Treatment regimen included the 5 most common regimens in the first- or second-line setting: FOLFOX (folinic acid, fluorouracil, and oxaliplatin) or CAPOX (capecitabine and oxaliplatin) with or without bevacizumab, FOLFIRI (leucovorin [folinic acid], fluorouracil, and irinotecan) or CAPIRI (capecitabine and irinotecan) with or without bevacizumab, FOLFOXIRI plus bevacizumab, FOLFOX plus anti–endothelial growth factor receptor (EGFR) therapy, and FOLFIRI plus anti-EGFR therapy.

### Statistical Analysis

We estimated 1-, 2-, and 3-year survival using Kaplan-Meier methods, overall and by race and ethnicity and neighborhood-level SES; survival was compared using log-rank tests. Follow-up time was accrued from the date of mCRC diagnosis until date of death or December 31, 2022. We also used Cox proportional hazards models to examine associations between race and ethnicity and neighborhood-level SES and all-cause mortality, adjusting for age and year at diagnosis, sex, insurance, practice type, clinical trial enrollment, first-line treatment, *RAS* variant, *BRAF* variant, and microsatellite instability, and accounting for clustering by practice (130 clinics). We evaluated the proportional hazards assumption by including interaction terms of log(time) × race and ethnicity and log(time) × neighborhood-level SES. The assumption was violated for each racial or ethnic group. Therefore, the adjusted hazards ratios indicate average hazard over the follow-up period.

All analyses were performed using SAS version 9.4 (SAS Institute). All tests were 2-sided, with *P* < .05 considered statistically significant.

## Results

We identified 3115 young adults diagnosed with mCRC (mean [SD] age at diagnosis, 42.4 [5.9] years; 1651 male [53.0%]; 122 Asian [3.9%], 424 Black [13.6%], 395 Hispanic [12.7%], 1874 White [60.2%], 300 other [9.6%]). Most patients were diagnosed with colon (vs rectal) cancer (2200 [70.6%] vs 864 [27.7%]). Patients initiated treatment a median (IQR) 55 (19-59) days from mCRC diagnosis. Prevalence of *RAS* variants, *BRAF* variants, and microsatellite instability was 42.2% (1315 patients), 4.6% (144 patients), and 5.4% (168 patients), respectively. First-line treatment regimens included FOLFOX/CAPOX with or without bevacizumab (1686 [54.1%]), FOLFIRI/CAPIRI with or without bevacizumab (469 [15.1%]), FOLFOXIRI with bevacizumab (218 [7.0%]), FOLFOX with anti-EGFR therapy (100 [3.2%]), and FOLFIRI with anti-EGFR therapy (82 [2.6%]).

Sociodemographic, tumor, and treatment characteristics differed by race and ethnicity (Table 1). For example, 1216 of 1874 White patients (64.9%) had private insurance compared with 239 of 424 Black (56.4%) and 209 of 395 Hispanic patients (52.9%), and clinical trial enrollment was lower among Hispanic (22 [5.6%]) and Asian (7 [5.7%]) patients compared with White patients (226 [12.1%]). There were also differences in these characteristics by neighborhood-level SES (Table 2). For example, a higher proportion of patients living in neighborhoods of the highest quintile of SES received care at academic settings (140 of 472 [29.7%]) and enrolled in a clinical trial (63 of 472 [13.4%]).

**Table 1.** Characteristics of Young Adults Diagnosed With Metastatic Colorectal Cancer by Race and Ethnicity, 2013-2021

Characteristics	Patients, No. (%) (N = 3115)				
	Asian (n = 122)	Black (n = 424)	Hispanic (n = 395)	White (n = 1874)	Other (n = 300)^a^
**Sociodemographic**					
Age at diagnosis, y					
18-29	6 (4.9)	21 (5.0)	23 (5.8)	67 (3.6)	16 (5.3)
30-39	23 (18.9)	92 (21.7)	85 (21.5)	398 (21.2)	66 (22.0)
40-49	93 (76.2)	311 (73.4)	287 (72.7)	1409 (75.2)	218 (72.7)
Sex^b^					
Male	65 (53.3)	201 (47.4)	189 (47.9)	1038 (55.4)	158 (52.7)
Female	57 (46.7)	223 (52.6)	206 (52.2)	836 (44.6)	142 (47.3)
Year of diagnosis^b^					
2013-2015	35 (28.7)	126 (29.7)	111 (28.1)	580 (31.0)	71 (23.7)
2016-2018	39 (32.0)	154 (36.3)	147 (37.2)	688 (36.7)	100 (33.3)
2019-2021	48 (39.3)	144 (34.0)	137 (34.7)	606 (32.3)	129 (43.0)
Insurance^b^					
Private or commercial	74 (60.7)	239 (56.4)	209 (52.9)	1216 (64.9)	167 (55.7)
Medicaid	13 (10.7)	44 (10.4)	47 (11.9)	135 (7.2)	32 (10.7)
Medicare	3 (2.5)	14 (3.3)	6 (1.5)	50 (2.7)	7 (2.3)
Other government	3 (2.5)	18 (4.3)	8 (2.0)	41 (2.2)	13 (4.3)
Uninsured	6 (4.9)	49 (11.6)	42 (10.6)	154 (8.2)	37 (12.3)
Other or unknown	23 (18.9)	60 (14.2)	83 (21.0)	278 (14.8)	44 (14.7)
Neighborhood SES quintile^b^					
1	9 (7.4)	124 (29.3)	126 (31.9)	225 (12.0)	54 (18.0)
2	18 (14.8)	86 (20.3)	75 (19.0)	330 (17.6)	57 (19.0)
3	24 (19.7)	81 (19.1)	57 (14.4)	367 (19.6)	61 (20.3)
4	32 (26.2)	66 (15.6)	53 (13.4)	433 (23.1)	54 (18.0)
5	31 (25.4)	23 (5.4)	31 (7.9)	349 (18.6)	38 (12.7)
Missing	8 (6.6)	44 (10.4)	53 (13.4)	170 (9.1)	36 (12.0)
Practice setting^b^					
Academic	16 (13.1)	62 (14.6)	61 (15.4)	366 (19.5)	21 (7.0)
Community	106 (86.9)	362 (85.4)	334 (84.6)	1508 (80.5)	279 (93.0)
**Tumor**					
Time from diagnosis to treatment, mean (SD), d^c^	61.0 (81.8)	58.5 (69.4)	56.3 (64.1)	52.7 (63.1)	51.8 (62.7)
Diagnosis type					
Recurrent metastasis	40 (32.8)	110 (25.9)	115 (29.1)	486 (25.9)	94 (31.3)
De novo metastasis	79 (64.8)	304 (71.7)	262 (66.3)	1355 (72.3)	196 (65.3)
Unknown or missing	3 (2.5)	10 (2.4)	18 (4.6)	33 (1.8)	10 (3.3)
Location^b^					
Colon	75 (61.5)	345 (81.4)	274 (69.4)	1293 (69.0)	213 (71.0)
Rectum	44 (36.1)	72 (17.0)	113 (28.6)	555 (29.6)	80 (26.7)
Unspecified	3 (2.5)	7 (1.7)	8 (2.0)	26 (1.4)	7 (2.3)
*RAS* variation^b^					
Yes	54 (44.3)	217 (51.2)	156 (39.5)	768 (41.0)	120 (40.0)
No	64 (52.5)	191 (45.1)	209 (52.9)	1025 (54.7)	162 (54.0)
Missing	4 (3.3)	16 (3.8)	30 (7.6)	81 (4.3)	18 (6.0)
*BRAF* variation^b^					
Yes	3 (2.5)	18 (4.3)	13 (3.3)	98 (5.2)	12 (4.0)
No	115 (94.3)	390 (92.0)	352 (89.1)	1695 (90.5)	270 (90.0)
Missing	4 (3.3)	16 (3.8)	30 (7.6)	81 (4.3)	18 (6.0)
Microsatellite instability					
Yes	11 (9.0)	21 (5.0)	22 (5.6)	96 (5.1)	18 (6.0)
No	107 (87.7)	387 (91.3)	343 (86.8)	1697 (90.6)	264 (88.0)
Missing	4 (3.3)	16 (3.8)	30 (7.6)	81 (4.3)	18 (6.0)
*KRAS*/*NRAS*/*BRAF* wild-type^b^					
Yes	35 (28.7)	91 (21.5)	97 (24.6)	581 (31.0)	89 (29.7)
No	83 (68.0)	317 (74.8)	268 (67.9)	1212 (64.7)	193 (64.3)
Missing	4 (3.3)	16 (2.8)	30 (7.6)	81 (4.3)	18 (6.0)
Lynch syndrome					
Yes	8 (6.6)	15 (3.5)	14 (3.5)	68 (3.6)	11 (3.7)
No	83 (68.0)	282 (66.5)	239 (60.5)	1200 (64.0)	194 (64.7)
Missing	31 (25.4)	127 (30.0)	142 (36.0)	606 (32.3)	95 (31.7)
**Treatment^d^**					
Clinical trial enrollment^b^					
Yes^e^	7 (5.7)	33 (7.8)	22 (5.6)	226 (12.1)	22 (7.3)
No	115 (94.3)	391 (92.2)	373 (94.4)	1648 (87.9)	278 (92.7)
First-line treatment					
FOLFOX or CAPOX with or without bevacizumab	69 (56.6)	245 (57.8)	208 (52.7)	1011 (54.0)	153 (51.0)
FOLFIRI or CAPIRI with or without bevacizumab	16 (13.1)	75 (17.7)	59 (14.9)	271 (14.5)	48 (16.0)
FOLFOXIRI with bevacizumab	9 (7.4)	21 (5.0)	24 (6.1)	141 (7.5)	23 (7.7)
FOLFOX with anti-EGFR therapy	1 (0.8)	12 (2.8)	16 (4.1)	65 (3.5)	6 (2.0)
FOLFIRI with anti-EGFR therapy	6 (4.9)	7 (1.7)	10 (2.5)	50 (2.7)	9 (3.0)
Second-line treatment (n = 1967)^f^					
FOLFOX or CAPOX with or without bevacizumab	17 (37.0)	38 (23.6)	49 (31.8)	169 (23.2)	25 (22.5)
FOLFIRI or CAPIRI with or without bevacizumab	21 (45.7)	96 (59.6)	80 (52.0)	393 (54.0)	60 (54.1)
FOLFOXIRI with bevacizumab	0	5 (3.1)	2 (1.3)	38 (5.2)	4 (3.6)
FOLFOX with anti-EGFR therapy	0	5 (3.0)	3 (2.0)	28 (3.9)	7 (6.3)
FOLFIRI with anti-EGFR therapy	8 (17.4)	17 (10.6)	20 (13.0)	100 (13.7)	15 (13.5)

Abbreviations: CAPIRI, capecitabine and irinotecan; CAPOX, capecitabine and oxaliplatin; EGFR, endothelial growth factor receptor; FOLFIRI, leucovorin (folinic acid), fluorouracil, and irinotecan; FOLFOX, folinic acid, fluorouracil, and oxaliplatin; FOLFOXIRI, folinic acid, fluorouracil, oxaliplatin, and irinotecan; SES, socioeconomic status.

^a^ Non-Hispanic other race included American Indian or Alaska Native, Hawaiian or Pacific Islander, and multirace individuals.

^b^ Statistically significant (*P* < .05) using a χ^2^ or Fisher exact test.

^c^ ANOVA *P* value = .29.

^d^ Five most common treatments received.

^e^ Enrollment in any line of treatment.

^f^ A total of 1148 patients (36.9%) did not receive second-line treatment and were excluded from percentages.

**Table 2.** Characteristics of Young Adults Diagnosed With Metastatic Colorectal Cancer by Neighborhood-Level SES, 2013-2021^a^

Characteristics	Patients, No. (%) (N = 2804)				
	Quintile 1 (n = 538)	Quintile 2 (n = 566)	Quintile 3 (n = 590)	Quintile 4 (n = 638)	Quintile 5 (n = 472)
**Sociodemographic**					
Age at diagnosis, y					
18-29	26 (4.8)	21 (3.7)	24 (4.1)	29 (4.6)	19 (4.0)
30-39	117 (21.8)	116 (20.5)	122 (20.7)	141 (22.1)	99 (21.0)
40-49	395 (73.4)	429 (75.8)	444 (75.3)	468 (73.4)	354 (75.0)
Sex					
Male	283 (52.6)	299 (52.8)	310 (52.5)	338 (53.0)	253 (53.6)
Female	255 (47.4)	267 (47.2)	280 (47.5)	300 (47.0)	219 (46.4)
Race and ethnicity^b^					
Asian	9 (1.7)	18 (3.2)	24 (4.1)	32 (5.0)	31 (6.6)
Black	124 (23.1)	86 (15.2)	81 (13.7)	66 (10.3)	23 (4.9)
Hispanic	126 (23.4)	75 (13.3)	57 (9.7)	53 (8.3)	31 (6.6)
White	225 (41.8)	330 (58.3)	367 (62.2)	433 (67.9)	349 (73.9)
Other^c^	54 (10.0)	57 (10.1)	61 (10.3)	54 (8.5)	38 (8.1)
Year of diagnosis					
2013-2015	149 (27.7)	149 (26.3)	162 (27.5)	202 (31.7)	141 (29.9)
2016-2018	189 (35.1)	224 (39.6)	214 (36.3)	220 (34.5)	175 (37.1)
2019-2021	200 (37.2)	193 (34.1)	214 (36.3)	216 (33.9)	156 (33.1)
Insurance^b^					
Private or commercial	310 (57.6)	330 (58.3)	360 (61.0)	420 (65.8)	338 (71.6)
Medicaid	77 (14.3)	58 (10.3)	50 (8.5)	34 (5.3)	16 (3.4)
Medicare	20 (3.7)	14 (2.5)	15 (2.5)	18 (2.8)	6 (1.3)
Other government	11 (2.0)	17 (3.0)	12 (2.0)	21 (3.3)	13 (2.8)
Uninsured	49 (9.1)	74 (13.1)	56 (9.5)	51 (8.0)	38 (9.1)
Other or unknown	71 (13.2)	73 (12.9)	97 (16.4)	94 (14.7)	61 (12.9)
Practice setting^b^					
Academic	58 (10.8)	67 (11.8)	95 (16.1)	131 (20.5)	140 (29.7)
Community	480 (89.2)	499 (88.2)	495 (83.9)	507 (79.5)	332 (70.3)
**Tumor**					
Time from diagnosis to treatment, mean (SD), d^d^	57.6 (65.7)	53.4 (59.2)	50.4 (65.1)	54.3 (68.2)	54.6 (7.4)
Diagnosis type					
Recurrent metastasis	147 (27.3)	170 (30.0)	174 (29.5)	153 (24.0)	130 (27.5)
De novo metastasis	368 (68.4)	384 (67.8)	407 (69.0)	473 (74.1)	333 (70.6)
Unknown or missing	23 (4.3)	12 (2.1)	9 (1.5)	12 (1.9)	9 (1.9)
Location					
Colon	380 (70.6)	381 (67.3)	425 (72.0)	448 (70.2)	327 (69.3)
Rectum	148 (27.5)	177 (31.3)	152 (25.8)	182 (28.5)	135 (28.6)
Unspecified	10 (1.9)	8 (1.4)	13 (2.2)	8 (1.3)	10 (2.1)
*RAS* variant					
Yes	225 (41.8)	233 (41.2)	245 (41.5)	277 (43.4)	203 (43.0)
No	280 (52.0)	309 (54.6)	314 (53.2)	338 (53.0)	257 (54.5)
Missing	33 (6.1)	24 (4.2)	31 (5.3)	23 (3.6)	12 (2.5)
*BRAF* variant^b^					
Yes	14 (2.6)	16 (2.8)	29 (4.9)	42 (6.6)	24 (5.1)
No	491 (91.3)	526 (92.9)	530 (89.8)	573 (89.8)	536 (92.4)
Missing	33 (6.1)	24 (4.2)	31 (5.3)	23 (3.6)	12 (2.5)
Microsatellite instability					
Yes	33 (6.1)	31 (5.5)	28 (4.8)	30 (4.7)	33 (7.0)
No	472 (87.7)	511 (90.3)	531 (90.0)	585 (91.7)	427 (90.5)
Missing	33 (6.1)	24 (4.2)	31 (5.3)	23 (3.6)	12 (2.5)
*KRAS*/*NRAS*/*BRAF* wild-type					
Yes	143 (26.6)	173 (30.6)	170 (28.8)	182 (28.5)	154 (32.6)
No	362 (67.3)	369 (65.2)	389 (65.9)	433 (67.9)	306 (64.8)
Missing	33 (6.1)	24 (4.2)	31 (5.3)	23 (3.6)	12 (2.5)
Lynch syndrome					
Yes	18 (3.4)	21 (3.7)	22 (3.7)	18 (2.8)	27 (5.7)
No	320 (59.5)	376 (66.4)	371 (62.9)	424 (66.5)	337 (71.4)
Missing	200 (37.2)	169 (29.9)	197 (33.4)	196 (30.7)	108 (22.9)
**Treatment^e^**					
Clinical trial enrollment^b^					
Yes^f^	37 (6.9)	45 (8.0)	59 (10.0)	83 (13.0)	63 (13.4)
No	501 (93.1)	521 (92.0)	531 (90.0)	555 (87.0)	409 (86.7)
First-line treatment					
FOLFOX or CAPOX with or without bevacizumab	300 (55.8)	281 (49.7)	317 (53.7)	344 (53.9)	253 (53.6)
FOLFIRI or CAPIRI with or without bevacizumab	89 (16.5)	94 (16.6)	90 (15.3)	86 (13.5)	74 (15.7)
FOLFOXIRI with bevacizumab	34 (6.3)	38 (6.7)	37 (6.3)	49 (7.7)	40 (8.5)
FOLFOX with anti-EGFR therapy	9 (1.7)	19 (3.4)	20 (3.4)	27 (4.2)	17 (3.6)
FOLFIRI with anti-EGFR therapy	12 (2.2)	19 (3.4)	17 (2.9)	18 (2.8)	10 (2.1)
Second-line treatment (n = 1785)^g^					
FOLFOX or CAPOX with or without bevacizumab	53 (23.4)	62 (25.8)	68 (25.2)	83 (29.3)	32 (17.8)
FOLFIRI or CAPIRI with or without bevacizumab	124 (54.6)	134 (55.8)	147 (54.4)	137 (48.4)	108 (60.0)
FOLFOXIRI with bevacizumab	7 (3.1)	10 (4.2)	10 (3.7)	13 (4.6)	9 (5.0)
FOLFOX with anti-EGFR therapy	9 (4.0)	8 (3.3)	10 (3.7)	10 (3.5)	6 (3.3)
FOLFIRI with anti-EGFR therapy	34 (15.0)	26 (10.8)	35 (13.0)	40 (14.1)	25 (13.9)

Abbreviations: CAPIRI, capecitabine and irinotecan; CAPOX, capecitabine and oxaliplatin; EGFR, endothelial growth factor receptor; FOLFIRI, leucovorin (folinic acid), fluorouracil, and irinotecan; FOLFOX, folinic acid, fluorouracil, and oxaliplatin; FOLFOXIRI, folinic acid, fluorouracil, oxaliplatin, and irinotecan; SES, socioeconomic status.

^a^ A total of 311 patients (10.0%) were missing SES information.

^b^ Statistically significant (*P* < .05) using a χ^2^ or Fisher exact test.

^c^ Non-Hispanic other included American Indian or Alaska Native, Hawaiian or Pacific Islander, and multirace individuals.

^d^ ANOVA *P* value = .47.

^e^ Five most common treatments received.

^f^ Enrollment in any line of treatment.

^g^ A total of 1019 patients (36.3%) did not receive second-line treatment and were excluded from percentages.

Survival differed by race and ethnicity and by neighborhood-level SES (Figure). For example, Black patients had the lowest 3-year survival (40.7%; 95% CI, 35.7%-45.6%) followed by patients categorized as other race (45.1%; 95% CI, 38.7%-51.3%), White (46.9%; 95% CI, 44.5%-49.3%), Hispanic (53.3%; 95% CI, 48.0%-58.3%), and Asian (57.5%; 95% CI, 47.9%-66.0%) patients (Table 3). This pattern was similar for 1- and 2-year survival. By neighborhood-level SES, patients living in the lowest compared with the highest quintile had worse 1-, 2-, and 3-year survival. For example, 3-year survival was 40.6% (95% CI, 36.1%-45.1%) among patients living in the lowest quintile compared to 58.9% (95% CI, 54.1%-63.4%) among those living in the highest quintile.

**Figure.**
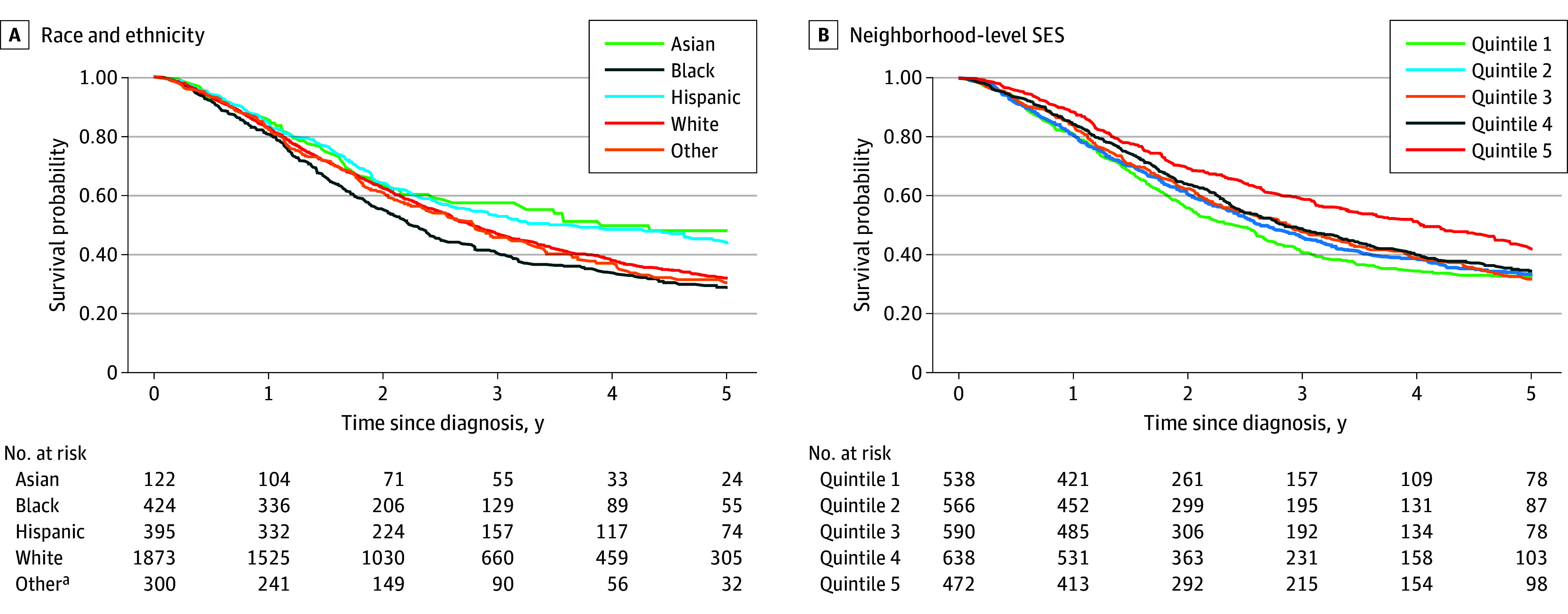
Kaplan-Meier Curves of Survival by Race and Ethnicity and Neighborhood-Level SES SES indicates socioeconomic status. ^a^Non-Hispanic other race included American Indian or Alaska Native, Hawaiian or Pacific Islander, and multirace individuals.

**Table 3.** Survival by Race and Ethnicity and Neighborhood-Level SES

Characteristics	1-y Survival, % (95% CI)	2-y Survival, % (95% CI)	3-y Survival, % (95% CI)
Race and ethnicity			
Asian	0.86 (0.79-0.91)	0.64 (0.55-0.72)	0.58 (0.48-0.66)
Black	0.81 (0.77-0.84)	0.55 (0.50-0.60)	0.41 (0.36-0.46)
Hispanic	0.85 (0.81-0.88)	0.64 (0.59-0.69)	0.53 (0.48-0.58)
White	0.83 (0.81-0.85)	0.63 (0.60-0.65)	0.47 (0.45-0.49)
Other^a^	0.83 (0.78-0.86)	0.61 (0.54-0.66)	0.45 (0.39-0.51)
Neighborhood-level SES quintile			
1	0.81 (0.78-0.84)	0.56 (0.51-0.60)	0.41 (0.36-0.45)
2	0.81 (0.77-0.84)	0.61 (0.56-0.65)	0.46 (0.41-0.50)
3	0.84 (0.81-0.87)	0.62 (0.58-0.66)	0.48 (0.43-0.52)
4	0.85 (0.81-0.87)	0.64 (0.60-0.68)	0.49 (0.45-0.53)
5	0.89 (0.85-0.91)	0.69 (0.65-0.73)	0.59 (0.54-0.63)

Abbreviation: SES, socioeconomic status.

^a^ Non-Hispanic other race included American Indian or Alaska Native, Hawaiian or Pacific Islander, and multirace individuals.

In adjusted models, the hazard ratios (HRs) for the association between race and ethnicity and all-cause mortality were 1.08 (95% CI, 0.90-1.31) for Black patients, 0.75 (95% CI, 0.53-1.08) for Asian patients, 0.82 (95% CI, 0.65-1.01) for Hispanic patients, and 0.89 (95% CI, 0.68-1.18) for other (vs White patients) (Table 4). Compared with those living in the highest quintile of neighborhood-level SES, patients living in the lowest quintile had statistically significant higher risk of mortality (Q1 vs Q5: HR, 1.51; 95% CI, 1.24-1.82).

**Table 4.** Adjusted Hazard Ratios for Race and Ethnicity and Neighborhood-Level SES

Characteristic	Adjusted HR (95% CI)^a^
Race and ethnicity	
Asian	0.75 (0.53-1.08)
Black	1.08 (0.90-1.31)
Hispanic	0.81 (0.65-1.01)
Other^b^	0.89 (0.68-1.18)
Neighborhood-level SES quintile	
1	1.51 (1.24-1.82)
2	1.29 (1.04-1.60)
3	1.16 (0.94-1.43)
4	1.19 (0.99-1.42)

Abbreviations: HR, hazard ratio; SES, socioeconomic status.

^a^ Adjusted for age and year at diagnosis, sex, insurance, practice type, clinical trial enrollment, first-line treatment, *RAS* variant, *BRAF* variant, and microsatellite instability.

^b^ Non-Hispanic other included American Indian or Alaska Native, Hawaiian or Pacific Islander, and multirace individuals.

## Discussion

In this diverse, community-based sample of young adults with mCRC, Black patients had worse 1-, 2-, and 3-year survival, while Asian and Hispanic patients had better survival compared with White patients. Patients living in lower SES neighborhoods also had worse survival compared with patients living in the highest quintile of neighborhood-level SES. Results were consistent in models adjusted for sociodemographic, tumor, and treatment characteristics: the lowest quintile of neighborhood-level SES was associated with all-cause mortality.

Our study adds further evidence to the well-documented racial and ethnic disparities among patients with colorectal cancer, particularly for those with metastatic disease.^3,17,18,19^ For example, an analysis of data from the SEER Program demonstrated that Black patients with early-onset mCRC had worse overall survival compared with White patients.^18^ In our study, we observed differences in 1-, 2-, and 3-year survival between Black and White patients with early-onset mCRC and although not statistically significant, the risk of all-cause mortality among Black patients was elevated in adjusted models. Our results can be interpreted through an integrated social determinants framework for cancer equity, in which inequities result from upstream structural conditions.^20^ These conditions encompass structural inequities and social injustice, institutional environments, and individuals’ living environments and create downstream consequences that affect factors such as health behaviors, health care utilization, and outcomes across the cancer continuum. For example, prior studies have linked racial residential segregation with colorectal cancer outcomes, wherein Black patients with colorectal cancer living in more segregated counties were more likely to present with advanced stage disease and have worse cancer-specific survival.^21^ Other downstream factors such as health insurance and quality of care may contribute to our findings and explain why Black-White survival disparities are particularly worse for distant stage colorectal cancer compared to localized stage. Differences in receipt of novel therapies, access to and enrollment in clinical trials, and response to therapy may also contribute to this disparity given the importance of treatment for patients with mCRC.^19,22^

Asian and Hispanic patients with mCRC in our study had better 1-, 2-, and 3-year survival compared with White patients and, although not statistically significant, the risk of all-cause mortality among Asian and Hispanic patients was lower in adjusted models. Previous studies of survival among Asian and Hispanic patients have reported mixed results.^3,17,23^ Some studies of patients with early-onset colorectal cancer (all stages) found that Asian and Hispanic patients had lower relative survival compared with White patients, while other studies have found that Asian and Hispanic patients have similar or better survival compared with White patients.^3,17^ Our finding may be explained by heterogenous Asian and Hispanic populations that comprise different acculturation experiences, ethnicities, and nativity status. For example, what has been called the “Hispanic paradox”—ie, the survival advantage among Hispanic patients despite being an underserved population—has been observed for many health outcomes including cancer, cardiovascular disease, and diabetes.^24^ The paradox may be explained by protective behaviors of foreign-born Hispanic patients, such as healthy diets, physical activity, and social support compared with US-born immigrants.^24^ In addition to acculturation, the heterogeneity of Asian and Hispanic populations may explain mixed results of previous studies and mask disparities in mCRC survival between disaggregated groups. In the US, the Asian race category homogenizes ethnicities of 49 countries, and the Hispanic ethnicity category homogenizes ethnicities of 19 countries and territories.^25^ A 2024 study^23^ that examined early-onset colorectal cancer survival by disaggregated Asian American groups found that Southeast Asian (HR, 1.10; 95% CI, 0.96-1.26) and Korean (HR, 1.10; 95% CI, 0.90-1.35) individuals had HRs greater than 1 for mortality compared with White individuals, while Chinese individuals had an HR lower than 1 (HR, 0.90; 95% CI, 0.78-1.04). In our study, the survival advantage among Asian patients may be explained by higher proportions of certain ethnic groups such as Chinese patients. However, further research among disaggregated racial and ethnic groups is needed to identify heterogeneity across groups and to avoid erroneously masking disparities.

We found that neighborhood-level SES was associated with survival, whereby patients living in the lowest quintile of SES (vs highest quintile) had increased risk of mortality. Neighborhood-level SES may act as a proxy for structural racism and/or may independently contribute to survival, as demonstrated in prior studies.^7,8^ For example, in a 2024 meta-analysis,^7^ lower neighborhood-level SES was associated with increased incidence and mortality of colorectal cancer. These associations may be explained by disadvantaged economic, physical, and social conditions of neighborhoods, which impact access to resources necessary for health care and healthy behaviors.^7^ Neighborhood disadvantages and barriers such as lack of transportation, lack of academic practices and clinical trial sites, and poor social support may impact colorectal cancer care and prognosis, particularly for patients with mCRC.^8^

As highlighted by the social determinants framework for cancer equity, improving mCRC survival for historically disadvantaged populations requires a multilevel approach of interventions and policies that address fundamental causes and systemic drivers of inequity. At the policy level, initiatives focused on affordable housing, local economic development, and investment in community-based clinics are necessary to address the lack of resources and infrastructure in low-SES neighborhoods. At the health system level, a comprehensive, equity-focused approach that emphasizes timely diagnosis, access to care, and development of specialized care centers is critical. For example, the development of dedicated early-onset colorectal cancer centers, especially in low-SES neighborhoods, is essential to provide multidisciplinary and specialized care.^26^ These centers would address unique needs of young patients and are particularly important for disadvantaged groups with limited access to clinical trials and second opinions from multidisciplinary teams. In community-based cancer clinics, expanding clinical trial access and enrollment is also essential to reducing survival disparities in mCRC.

### Strengths and Limitations

Strengths of our study include the diverse, community-based sample and the detailed electronic health record information that allowed us to adjust for tumor and treatment characteristics. Our findings are unlikely explained by differences in screening uptake because screening had not yet been recommended for this younger age group. Similarly, it is unlikely that differential receipt of treatment explains our findings because there were no differences in time to treatment or treatment regimens by race and ethnicity and neighborhood-level SES.

This study also had several limitations. Asian, Hispanic, and other race groups were aggregated and may not reflect or be generalizable to disaggregated groups. In addition, the sample size was small for Asian patients, and estimates were therefore imprecise. Finally, we evaluated all-cause mortality because Flatiron Health does not provide information on cause of death. However, all-cause mortality is a reasonable approximation of cancer-specific mortality because all patients had metastatic disease and other causes of death are unlikely.

## Conclusions

In this cohort study of young adults with mCRC, survival was lower among Black patients and patients in the lowest SES quintile. However, only the association between neighborhood-level SES and survival remained statistically significant after adjusting for covariates. Our findings demonstrate the role of upstream structural conditions, such as inequitable distribution of resources in low-SES neighborhoods, that impact survival in this growing population and highlight the need for multilevel interventions and policies to reduce disparities.

## Notes

Supplement. Data Sharing Statement
