# Supplementary material for: Racial, Ethnic, and Socioeconomic Survival Disparities in Early-Onset Metastatic Colorectal Cancer
Source: JAMA Netw Open. 2026 Jan 8;9(1):e2553146. doi: 10.1001/jamanetworkopen.2025.53146 (PMC12784224; doi:10.1001/jamanetworkopen.2025.53146)
Supplement: Supplement. — Data Sharing Statement [file jamanetwopen-e2553146-s001.pdf]

Data Sharing Statement

Wang. Racial, Ethnic, and Socioeconomic Survival Disparities in Early-Onset Metastatic Colorectal Cancer. *JAMA Netw Open*. Published January 08, 2026.
doi:10.1001/jamanetworkopen.2025.53146

Data
Data available: No

Additional Information
Explanation for why data not available: The data underlying this article were provided by Flatiron Health by permission and will not be made available to other researchers due to this.
